# Impact of reduced dose schedule of PCV10 on pneumococcal carriage in Vietnam

**DOI:** 10.1056/NEJMoa2400007

**Published:** 2024-11-28

**Authors:** Lay-Myint Yoshida, Michiko Toizumi, Hien Anh Thi Nguyen, Billy J Quilty, Le Thuy Lien, Le Huy Hoang, Chihiro Iwasaki, Mizuki Takegata, Noriko Kitamura, Monica L Nation, Jason Hinds, Kevin van Zandvoort, Belinda D Ortika, Eileen M Dunne, Catherine Satzke, Hung Do Thai, Kim Mulholland, Stefan Flasche, Dang Duc Anh

**Affiliations:** 1. Nagasaki University, Institute of Tropical Medicine, Department of Pediatric Infectious Diseases, Nagasaki, Japan; 2. Nagasaki University, School of Tropical Medicine and Global Health, Department of Global Health, Nagasaki, Japan; 3. Nagasaki University Graduate School of Biomedical Science, Nagasaki, Japan; 4. National Institute of Hygiene and Epidemiology, Department of Bacteriology, Hanoi, Viet Nam; 5. London School of Hygiene & Tropical Medicine, Department of Infectious Disease Epidemiology, London, United Kingdom; 6. London School of Hygiene & Tropical Medicine, Centre for Mathematical Modelling of Infectious Diseases, London, United Kingdom; 7. Pasteur Institute in Nha Trang, Department of Bacteriology, Nha Trang, Viet Nam; 8. National Institute of Infectious Diseases, Tokyo, Japan; 9. Murdoch Children’s Research Institute, Infection and Immunity, Melbourne, ACT, Australia,; 10. The University of Melbourne, Department of Paediatrics, Melbourne, VIC, Australia,; 11. St Georges University, Institute for Infection and Immunity, London, United Kingdom; 12. The University of Melbourne, Peter Doherty Institute for Infection and Immunity, Department of Microbiology and Immunology, Parkville, Victoria, Australia; 13. Charite - Universitätmedizin Berlin, Centre for Global Health, Berlin, Germany

## Abstract

**BACKGROUND:**

We investigated the non-inferiority of a single priming and booster dose (1p+1) compared to 3 alternative dose schedules in sustaining control of PCV10 type carriage.

**METHODS:**

In PCV-naïve Nha Trang, Vietnam, a PCV10 catch-up campaign was offered to children <3 years old followed by a cluster randomized trial with four intervention arms (1p+1, 0p+1, 2p+1, 3p+0). Annual carriage surveys in infants and toddlers were conducted between 2016 and 2020. The primary endpoint was non-inferiority of 1p+1 in protecting against pneumococcal vaccine type (VT) carriage in infants/toddlers compared to 2p+1 and 3p+0 arms, 3.5 years after introduction.

**RESULTS:**

Overall VT carriage in infants in 2016 before PCV10 introduction in intervention arms was 160/1363 (11.7%) and in 2020 reduced to 6/333 (1.8%), 5/340 (1.5%), and 4/313 (1.3%) in 1p+1, 2p+1, and 3p+0 arms respectively: a non-inferior difference of 1p+1 against 2p+1 and 3p+0 of 0.3% (−1.6, 2.2%) and 0.5% (−1.4, 2.4%). Similarly, 1p+1 was found non-inferior for protection against VT carriage in toddlers. Serotype 6A carriage prevalence in infants was 99/1363 (7.3%) in 2016 and reduced to 12/333 (3.6%), 10/340 (2.9%) and 3/313 (1.0%) in 1p+1, 2p+1, and 3p+0 arms in 2020. Protection offered by 0p+1 was also non-inferior in infants and toddlers compared to three dose schedules, although cross protection against 6A was less prominent. No PCV-associated severe adverse effects were observed.

**CONCLUSIONS:**

The 1p+1 reduced dosing schedule of PCV10 was not inferior to alternative vaccine dosing schedules in protection against VT carriage in infants and toddlers.

(Funded by the Bill and Melinda Gates Foundation, and others; ClinicalTrials.gov number, NCT02961231.)

*Streptococcus pneumoniae* is a major cause of morbidity and mortality in children <5 years globally with most cases occurring in low and middle income countries (LMICs).^1,2^ Pneumococcal conjugate vaccines (PCVs) can prevent pneumococcal disease through direct and indirect protection by reduction of vaccine-types (VT) nasopharyngeal carriage.^3-7^ WHO recommends PCVs as either three primary doses given during early infancy (3p+0) or two primary doses given in early infancy and a booster dose from 9 months onward (2p+1).^8^ However, high vaccine costs have proven a barrier for introduction in many middle income countries and raise concerns for sustainability of the PCV program in LMICs who transition out of Gavi’s support for vaccine costs.

Following the control of pneumococcal vaccine-type disease and colonization through vaccination, a PCV schedule with a single priming and booster dose (1p+1) may be sufficient to sustain that control at reduced costs.^9^ Trials in England,^10^ South Africa,^11^ India^12^ and Vietnam^13^ with either immunogenicity or carriage outcomes have demonstrated that a 1p+1 schedule indeed induces a similar protection to a 2p+1 schedule following administration of the booster dose, however, they also confirmed suspected inferiority before the booster. While the similar post-booster direct protection against VT carriage is thought to sustain indirect protection in the first year of life, available evidence is lacking.^14,15^ Nasopharyngeal carriage is a pre-requisite for disease and reduction of carriage is an indicator of reduction of risk for disease as well as an indirect measure of herd immunity.^16^ Thus, measuring the impact of a vaccination schedule on carriage can be a proxy for measuring the impact of the schedule at a community level.

We conducted a cluster randomized non-inferiority trial to estimate the effect of PCV10 given in a reduced dosing schedule (1p+1 & 0p+1) compared to WHO recommended 2p+1 and 3p+0 schedule in a PCV naïve population in Vietnam. We here report whether the 1p+1 or 0p+1 schedules were non-inferior compared to the three dose schedules in maintaining control of VT carriage.

## METHODS

### STUDY DESIGN: cRCT

**Figure 1.**
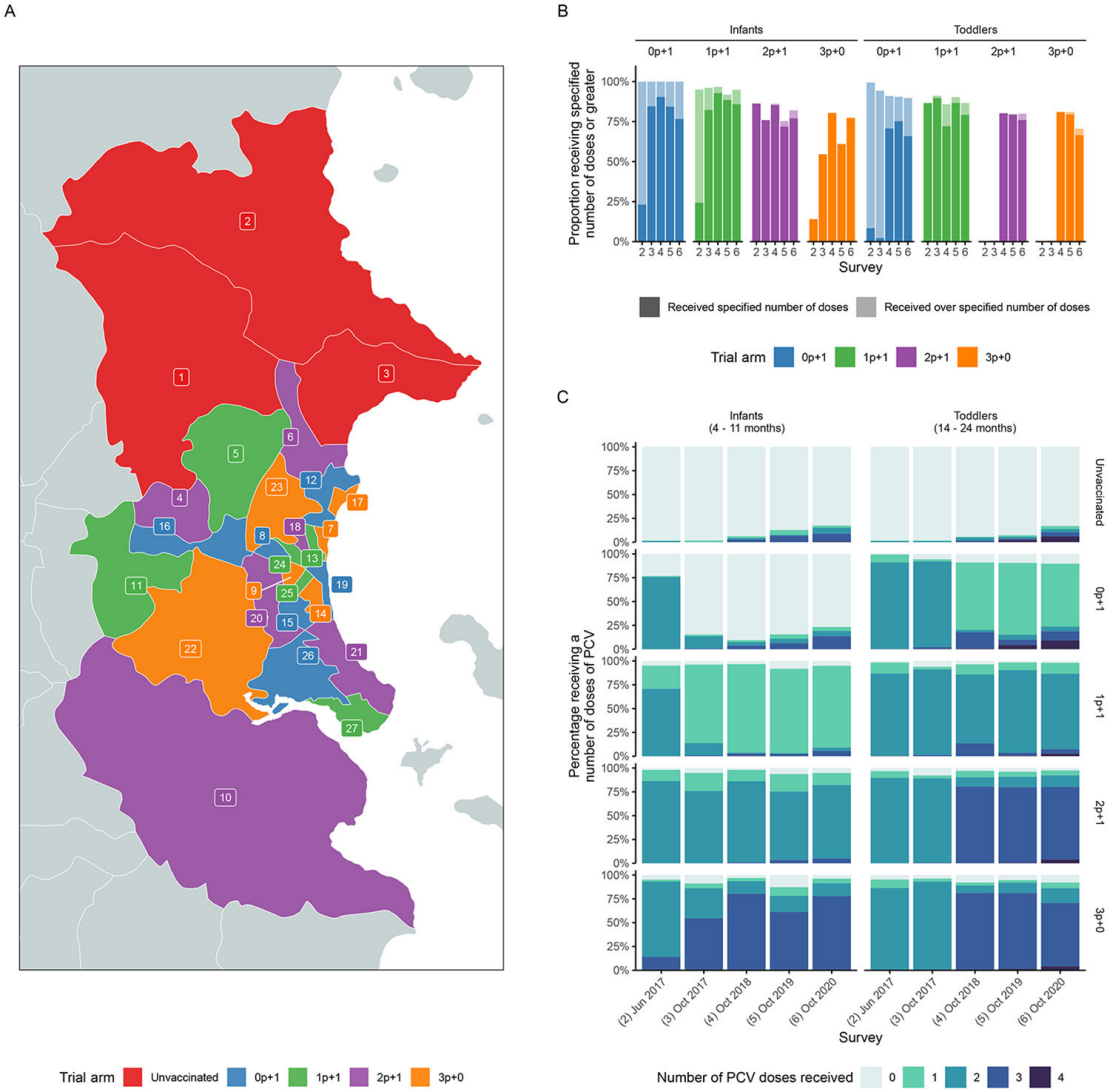
Study site map, proportion of population that received different PCV10 doses by age groups and arms Panel A shows the map of communes in Nha Trang with trial arm cluster allocation (unvaccinated, 0+1, 1p+1, 2p+1, 3p+0); panel B shows the proportion receiving the specified number of doses (dark section of bar) or greater (lighter section of bar) by trial arm and age group. Note that for the 0p+1 infant group, the specified number of doses is 0. Panel C shows the proportion reporting receiving different numbers of doses specified by trial arm and age group.

An open-label, non-inferiority, cluster randomized trial, was conducted across the 27 communes of Nha Trang city, south-central Vietnam. Communes were deemed the natural unit of cluster-randomization as they represent organization units for administration of different schedules in respective health centers and limit the risk for spill over due to the commune-based educational system. Three communes in the north were selected to remain with the current standard of care in Vietnam and not be included into the intervention part of the trial but monitored for changes in pneumococcal epidemiology in the trial area. The remaining 24 communes were randomized and assigned to four intervention arms/schedules (2p+1, 3p+0, 1p+1 and 0p+1). To ensure random but geographically balanced allocation of clusters that were similarly representative of rural and urban communities and that were included in the ongoing hospital-based pediatric ARI surveillance, we used automated rejection sampling as previously described (Figure 1A).^17^ The authors vouch for the accuracy and completeness of data and data analyses, along with the conduct of the trial according to the protocol (available at NEJM.org).

### INTERVENTION: PCV10

PCV10 (Synflorix^®^, GSK Vaccines) was used since it was the only PCV registered at the time of the study initiation in Vietnam. To accelerate indirect protection, in February 2017 a PCV catch-up vaccination campaign was conducted in the 24 intervention clusters to all children <3y old and eligible for national immunization vaccination (2-6 month: 3 doses, 7-18 month: 2 doses and 19-36 month: 1 dose respectively).

From March 2017 PCV10 was integrated into the national immunization program of the 24 intervention clusters according to their clinical trial designated PCV schedule. Children resident received PCV10 at 2, 3 and 4 months in the 3p+0 arm, at 2, 4 and 12 months in the 2p+1 arm, at 2 and 12 months in the 1p+1 arm and at 12 month in the 0p+1 arm.

### OUTCOME: VT carriage prevalence

Six carriage surveys; a pre-vaccination baseline carriage survey in all arms in October 2016, a post PCV10 catch-up carriage survey in June 2017 (5 months after the catch-up), and annual carriage surveys in November 2017 (7 months post-PCV), October 2018 (1.5 years post-PCV), 2019 (2.5 years post-PCV), and 2020 (3.5 years post-PCV) were conducted. All children eligible to receive routine immunization were included in the randomization. For each carriage survey, we aimed to recruit 60 children 4 to 11 months old (“infants”), and 60 children 14 to 24 months old (“toddlers”) from each commune.

Demographic information and nasopharyngeal samples were collected using standard procedures.^18^ DNA was extracted from the nasopharyngeal samples using a QiaCube HT instrument (QIAmp 96 DNA QIAcube HT Kit) and screened for *S.pneumoniae lytA* gene by realtime PCR at Pasteur Institute in Nha Trang.^19,20^ Positive samples were cultured, DNA extracted and transported to Murdoch Children’s Research Institute (MCRI) for serotype determination by microarray (Senti-SP v1.5, BUGS Bioscience, London, UK; https://bugsbio.org).

### STATISTICAL ANALYSES: changes in risk of VT carriage and non-inferiority

Sample size calculations deemed that with six clusters per intervention arm and 60 infants recruited per cluster we would have >80% power under a type I error probability of 5% to detect a at least five percentage point higher VTs carriage between a reduced dose arms/schedule and a three-dose arm/schedule with an assumed residual VTs carriage prevalence of 5%.^17^

PCV10-VTs carriage was defined as carriage of a serotype included in the PCV10 formulation (1, 4, 5, 6B, 7F, 9V, 14, 18C, 19F, or 23F). Due to the likely cross protection of PCV10 against 6A, we repeated analyses including 6A as a VT.

We calculated changes in the proportion of pneumococcal carriers who carry a VT, rather than carriage prevalence, due to the potential impact of COVID-19 control measures in 2020 on pneumococcal transmission and carriage. We used Poisson regression with a log offset for the number of positive samples to assess the change in the risk of VT carriage in both the post-catch-up survey and final carriage survey versus the baseline survey and tested for possible effect modification by age group and trial arm.

The primary endpoint of the trial was conducted as pre-specified: Non-inferiority was assessed by estimating the absolute difference in vaccine-type prevalence between two arms in the final carriage survey in October 2020. If the 95% confidence intervals (CIs) of the difference in VTs prevalence included no difference but not the 5% non-inferiority margin, the non-inferiority criteria was met.^21^ The margin was chosen so that non-inferiority would correspond to retention of at least 80% of the protective effect under the assumption that a 3 dose schedule would reduce VT prevalence from 32.5% to 5%.^17^ As a sensitivity analysis, we also assessed non-inferiority in the penultimate carriage survey in October 2019, just over 2.5 years after the start of routine vaccination according to the intervention schedules, but prior to the COVID-19 pandemic.

### ETHICAL APPROVAL

Ethical approval for the study was obtained from the Ministry of Health, Vietnam (4875/QD-BYT), and Nagasaki University (15120149). Written informed consent was obtained from the parents or guardian of the study participants at vaccination and sampling, and the study was conducted in accordance with the approved guidelines.

## RESULTS

### PCV VACCINATION COVERAGE

**Figure 2.**
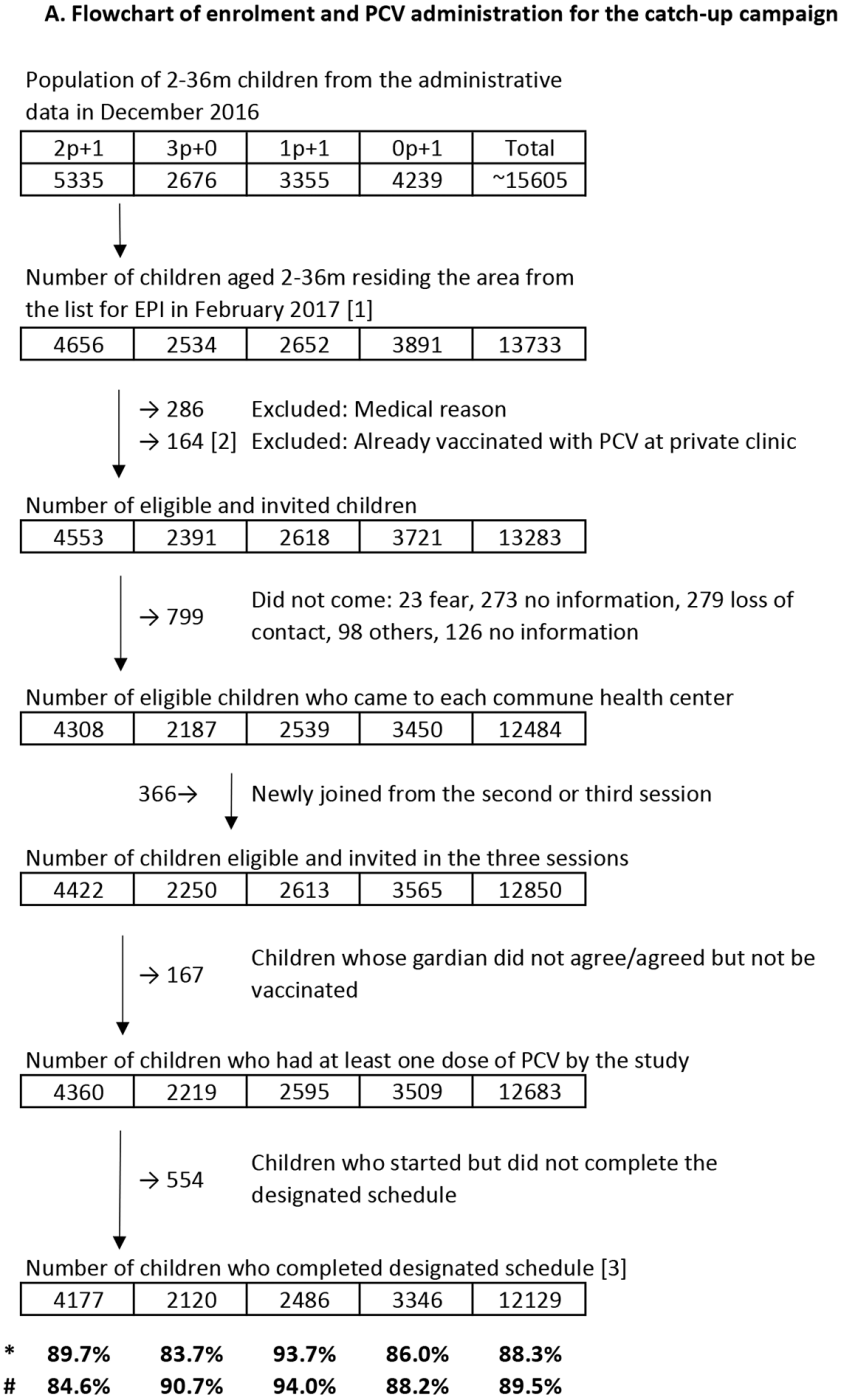

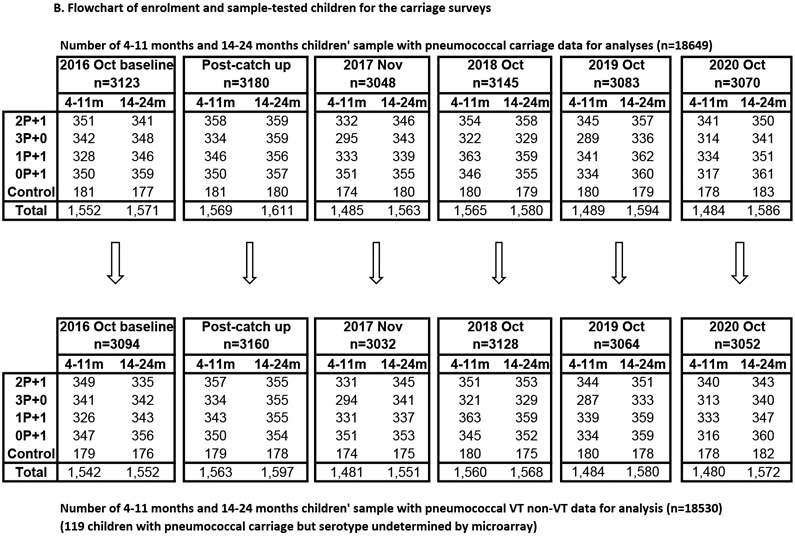
Flowchart of enrolment and sample-tested children for the carriage surveys, and enrolment and PCV administration for the catch-up campaign Panel A shows the number of children (infant: 4-11-month, toddler: 14–24-month-old) enrolled in each carriage survey. A total six carriage surveys; 1st: a pre-vaccination baseline carriage survey in all arms in 2016 October, 2nd: a post PCV-10 catchup carriage survey in June 2017 (5 months after the catch-up), and 3rd: annual carriage surveys in November 2017, 4th: October 2018, 5th: October 2019, and 6th: October 2020 were conducted. Panel B shows the number of children enrolled and the PCV10 vaccination coverage in the PCV10 catch-up campaign. *Completion rate (coverage of the catch-up campaign): ([3]/[1]) #Completion rate (coverage of PCV, assuming [2] completed a designated schedule): (([2]+[3])/(1))

For the catch-up vaccination, 13,733 children <3 years old residing in the intervention communes were age-eligible, and 12,850 children without precluding medical conditions or previous PCV vaccination were invited to receive PCV10 catch-up vaccination. With 20,434 doses given, 12,683 (98.7%) children received at least one dose of PCV10 and 12,129 (94.4%) completed the designated catch-up schedule and little differences in coverage between intervention arms were observed (Figure 2A).

As part of routine vaccination, 31,385 PCV10 doses were given to eligible children during the study period. In the survey in Oct 2020, >77.7% of infants and >70.7% of toddlers had received at least the number of doses specified for their age group and trial arm (Figure 1B, Table S2). There was some evidence of individuals reporting to have received additional doses to that specified by the trial schedule, perhaps through the private market (Figure 1B, Table S2).

### CHARACTERISTICS OF Participants: at baseline and subsequent carriage surveys

A total of 3124 children (2p+1: 692, 3p+0: 691, 1p+1: 674, 0p+1: 709, unvaccinated: 358) were enrolled in the baseline survey. The characteristics of the children are shown in table S1. Briefly, about 55% were male, more than 99% had received at least one dose of BCG and DTP and less than 2% had received at least one dose of PCV. Children were generally healthy with <2% reporting underlying medical conditions, albeit nearly half had mild respiratory symptoms in the preceding two weeks with more than 20% reported recent antibiotic use. The average household size was five. None of the socio-demographic characteristics differed across study arms (Table S1).

A total of 15,528 children were enrolled (post catch-up: 3181, four annual carriage surveys: 12,347), 15,526 (>99.9%) samples were determined for pneumococcal carriage and serotype result was determined for 15,436 (>99.0%) (Figure 2B).

### CHANGES IN PNEUMOCOCCAL CARRIAGE

**Figure 3.**
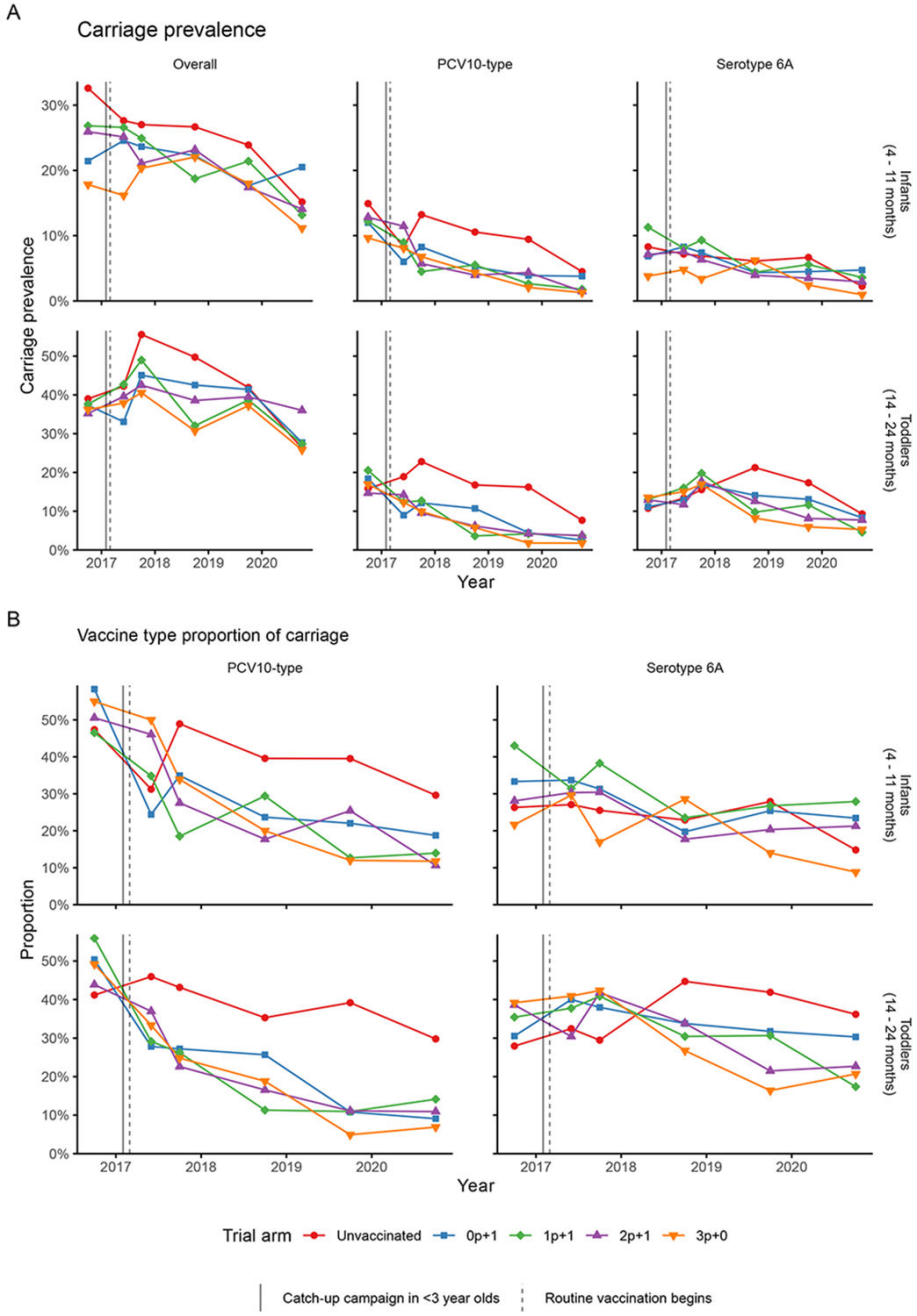
Pneumococcal carriage prevalence and vaccine type proportion of carriage in trial arms across carriage surveys Panel A shows the prevalence of carriage and panel B shows the proportion of carriage, by carriage survey (6 carriage surveys conducted between 2016 October to 2020 October), trial arm, age group, and vaccine type definition.

Overall pneumococcal carriage prevalence at baseline survey in 2016 in all intervention arms was 23.0% (315/1371) in infants and 36.6% (510/1394) in toddlers (Figure 3A, Table S3); of those carriers, 52.1% (160/307) in infants and 50.0% (246/492) in toddlers carried at least one serotype included in PCV10 (Figure 3B, Table S3) for a PCV10-type prevalence of 11.7% (160/1363) and 17.9% (246/1376), respectively. Serotype 6A was predominant with 32.2% (99/307) and 35.8% (176/492) in infants and toddlers, respectively. The major VTs at baseline were 19F, 6B, 23F, and 14 (Figure S2, Table S3).

In June 2017, 5 months after the catch-up campaign, 34.1% (291/854) of carriers carried at least one PCV10 type (infants: 37.7% (120/318), toddlers: 31.9% (171/536). The risk of a carrier carrying a PCV10 type were 32.9% lower (risk ratio, (RR): 0.67, 95% CI:0.58, 0.78 ) compared to the pre-PCV baseline in intervention clusters. There was no concomitant change in the risk of a carrier carrying serotype 6A after the catch-up campaign (34.4% (275/799) to 35.0% (299/854), RR: 1.02, 95% CI: 0.86, 1.2 ). No evidence was found of effect modification on the change in risk by age group or schedule (with 2p+1 as reference) for either PCV10 or serotype 6A.

Compared to the pre-PCV baseline, by October 2020 (>3.5 years post-PCV introduction), 11.6% (68/585) of carriers carried at least one PCV10 type (infants: 14.4% (27/188), toddlers: 10.3% (41/397)). The risk of a carrier carrying a PCV10 type were 77.1% (RR: 0.23, 95% CI: 0.18, 0.29) lower across intervention clusters. A 34.9% reduction in the risk of carriage being serotype 6A was also observed (in 2020: 22.4% (131/585), RR: 0.65, 95% CI: 0.53, 0.80). No evidence was found of effect modification by age group or schedule (when 2p+1 was set as the reference category). The proportion of carriers carrying major PCV10 types was reduced (Figure S2, Table S3).

### NON-INFERIORITY OF REDUCED DOSE SCHEDULES

**Figure 4.**
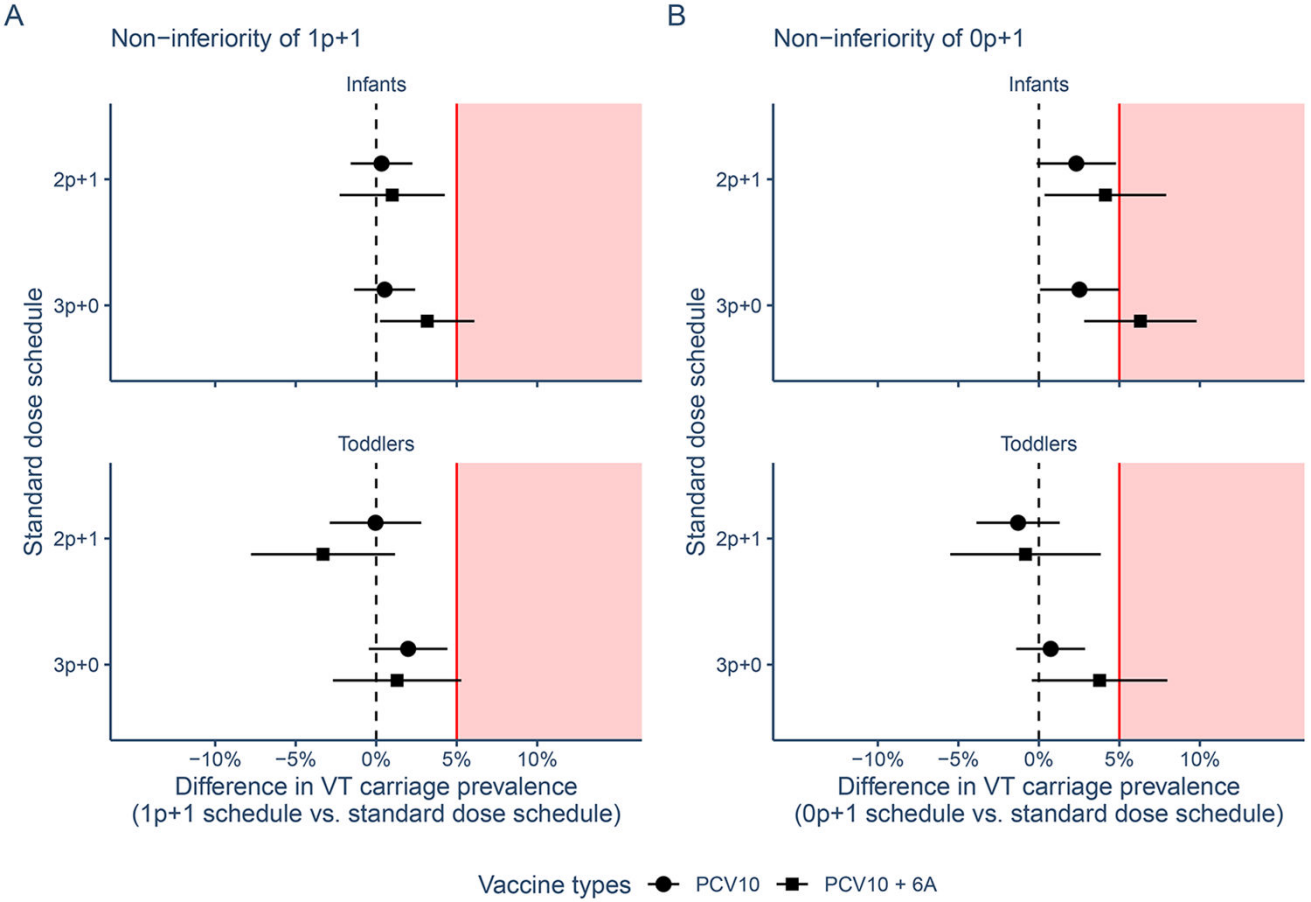
Non-inferiority analysis 1p+1 and 0p+1 arms versus 2p+1 and 3p+0 arms The figure shows the non-inferiority of reduced dose schedules (panel A: 1p+1) and (panel B: 0p+1) versus standard dose schedules (2p+1 and 3p+0) by age group (infants: 4-11 months, toddlers: 14-24 months) by mean (point) and 95% confidence intervals (CI) (line) of the difference in absolute vaccine-type prevalence in the final carriage survey in October 2020 for PCV10 and PCV10 + serotype 6A. Red line and shaded area indicate the 5% non-inferiority margin; estimates with 95% CIs overlapping the 5% margin indicate inferiority.

In October 2020, 1p+1 was non-inferior to 2p+1 in maintaining control of PCV10 serotypes in infants: VT carriage prevalence was 0.3 percentage points (95% confidence interval (CI): −1.6, 2.2) higher in infants residing in the 1p+1 arm compared to those in the 2p+1 arm and thus below the pre-specified 5% margin. Similarly, for toddlers the difference was 0.0% (95% CI: −2.9, 2.8%). The 1p+1 schedule was also non-inferior to 3p+0 schedule in infants where VT carriage prevalence was 0.5% (95% CI: −1.4, 2.4%) higher and toddlers where it was 2.0% (95% CI: −0.5, 4.4%) higher (Figure 4A, Table S2).

The 0p+1 was also non-inferior to 2p+1 in infants with a 2.3% (95% CI: −0.1, 4.8%) higher VT carriage prevalence in Oct 2020. In toddlers VT prevalence was 1.3% lower (−1.3% difference, 95% CI: −3.9, 1.3%). The 0p+1 schedule was similarly non-inferior to 3p+0 in infants and toddlers (Figure 4B, Table S2).

With the inclusion of 6A as a VT the 1p+1 schedule remained non-inferior to 2p+1 but not to 3p+0. The 0p+1 schedule only remained non-inferior to 2p+1 in toddlers (Figure S1, Table S2).

These results did not change qualitatively when assessing non-inferiority in the last pre-pandemic year of the trial (in October 2019 survey), i.e. just over 2.5 years after the start of PCV10 vaccination with the exception of 1p+1 and 0p+1 being inferior to 2p+1 and 3p+0 in toddlers when including 6A as a VT (Figure S1).

### SAFETY

During the study period, 49 (including 7 after catch-up campaign) serious adverse events were reported within 28 days of vaccination: 0.09% out of 51,819 doses given. After review by an independent panel, this panel deemed that none were thought to be related to PCV10 vaccination.

## DISCUSSION

We report the findings of a cluster-randomized PCV10 reduced-dosing schedule trial. We found that PCV10 reduced-dose schedule (1p+1 or 0p+1) maintained combined direct and indirect protection against VT carriage similarly to 3-dose schedules.

Results from individually randomized reduced-dosing schedules trials in the UK, South Africa, India, and Vietnam have consistently shown non-inferiority of the post-booster response of 1p+1 across all three currently WHO recommended PCV products.^7-10^ Similarly, studies from India^22^ and Vietnam^23,24^ have shown high-efficacy against vaccine serotype carriage in the second year of life following a reduced-dose schedule with either PCV10 or PCV13. While in the UK a 1p+1 schedule has been used since 2020,^25^ the COVID-19 pandemic has obscured any potential effects of increased risk for VT in infants thus far. We here show that in a setting with moderate pneumococcal carriage prevalence, where transmission usually concentrated in pre-school children, and high coverage, the direct protection of PCV against infection and transmission in 12 month olds can help to decrease VT circulation.

When serotype 6A was included as a PCV10 cross protective VT, 1p+1 remained noninferior to 2p+1 in both infants and toddlers but the non-inferiority criteria was no longer met in comparison with the 3p+0 schedule. This was also observed for the 0p+1 schedule. It is unclear why a schedule with 3 priming doses would elicit stronger protection against the circulation of cross protective 6A than all tested booster dose schedules, and we cannot rule out chance. Use of 6A-containing vaccines may be prioritized in settings, such as Vietnam, where 6A prevalence is high. Further investigations are required to provide additional evidence for the indirect protection against 6A in the different dosing schedules.

We included an explorative 0p+1 arm and found little evidence for its inferiority compared to three dose schedules. This adds further credibility to recent findings in that a single dose of PCV10 can elicit a reasonably strong immune response even in very young children^26^ and an estimated direct vaccine efficacy against VT carriage of about 50% following a single dose of PCV10 given at 12 months^24^. It also provides some evidence that single dose campaigns if delivered with high coverage in children as young as 12 months could help prevent a considerable burden in settings where multi dose administration may prove difficult.^27^

### LIMITATIONS

Due to COVID-19, non-pharmaceutical interventions were introduced in the study site between April and July 2020. As a result, pneumococcal carriage prevalence reduced by about 30% and decreased our power to detect a 5% absolute difference and detect potential inferiority. However, through annual surveys we showed consistent declines in VT prevalence before 2020 with a sensitivity analysis using 2019 data as primary endpoint which supports our findings (Figure S1). Owing to the single city setup of the trial there may have been some inter-cluster contamination which in principle could have obscured differences in vaccine effects between study arms. However, study clusters were deliberately chosen as administrative units that govern school attendance, the primary age groups for pneumococcal transmission in this setting,^17^ thus limiting contamination. Also, we included three unvaccinated communes, bordering the intervention arms and showed limited effects of PCV vaccination in the intervention arms on VT transmission in the unvaccinated communes. Long term suppression of VT carriage and serotype replacement by reduced dosing schedule should be monitored. Finally, it remains unclear if reliance on indirect protection is sufficient to sustain protection in settings with more intensive pneumococcal transmission, particularly in older age groups and with poorer booster dose coverage. The ongoing trial in the Gambia and mathematical modelling can help to explore the generalizability of our findings.

### CONCLUSION

In this cRCT in Vietnam, after more than 3.5 years of use at high coverage, a PCV10 1p+1 dosing schedule was non-inferior to 2p+1 or 3p+0 in infants and toddlers in controlling VT carriage in the community. The study results provide evidence for future PCV vaccination policy especially in LMICs.

## Supplementary Material

supplement

## Data Availability

In accordance with the Bill & Melinda Gates Foundation policy on open data access, data will be shared on request from the corresponding author on a collaborative basis. Personal information will be removed in the shared data.
