# Supplementary material for: Impact of reduced dose schedule of PCV10 on pneumococcal carriage in Vietnam
Source: N Engl J Med. Author manuscript; Available in PMC 2024 Dec 20. (PMC11661757; doi:10.1056/NEJMoa2400007)
Supplement: supplement [file NIHMS2017486-supplement-supplement.pdf]

Table of contents

Supplementary tables

Table S1. Characteristics of children participated in the first carriage survey by arm (pre-PCV)

Table S2: Proportion of children completing the specified schedule for age group and trial arm in the final cross-sectional survey (October 2020). For infants, this represents finishing the specified primary series and for toddlers, this represents finishing both the specified primary series + booster.

Table S3: Non-inferiority of reduced dose schedules (A: 1p+1, B: 0p+1) versus standard dose schedules (2p+1 and 3p+0) by age group (infants: 4-11 months, toddlers: 14-24 months) by mean and 95% confidence intervals (CI) of the difference in absolute prevalence in the penultimate carriage survey in 2019 for PCV-10 and PCV-10 + serotype 6A.

Table S4: PCV Reduced Dosing Schedules study subjects Severe Adverse Events list

Supplementary Figures

Figure S1: Non-inferiority results using 2019 vaccine type pneumococcal carriages.

Figure S2: Proportion of pneumococcal serotype carriage at baseline 2016 and 2020

Table S1. Characteristics of children participated in the first carriage survey by arm (pre-PCV)

Characteristics	2p+1 Number (%)	3p+0 Number (%)	1p+1 Number (%)	0p+1 Number (%)	Unvacciantied Number (%)
	n=692	n=691	n=674	n=709	n=358
**Demographic**					
Age group					
4-11	351 (50.7)	342 (49.5)	328 (48.7)	350 (49.4)	181 (50.6)
14-24	341 (49.3)	349 (50.5)	346 (51.3)	359 (50.6)	177 (49.4)
Sex					
Boys	373 (53.9)	340 (49.2)	365 (54.2)	373 (52.6)	197 (55.0)
Girls	319 (46.1)	351 (50.8)	309 (45.9)	336 (47.4)	161 (45.0)
**Perinatal & breastfeeding**					
Low birth weight (<2500 gram)					
Yes	18 (2.6)	14 (2.0)	19 (2.8)	12 (1.7)	6 (1.7)
No	674 (97.4)	677 (98.0)	655 (97.2)	697 (98.3)	352 (98.3)
Preterm born (gestational age at birth <37 weeks)					
Yes	39 (5.6)	28 (4.1)	40 (5.9)	49 (6.9)	16 (4.5)
No	653 (94.4)	663 (96.0)	634 (94.1)	660 (93.1)	342 (95.5)
Mode of delivery					
Vaginal	407 (58.8)	376 (54.4)	396 (58.8)	367 (51.8)	191 (53.4)
Cesarean section	285 (41.2)	315 (45.6)	278 (41.3)	342 (48.2)	167 (46.7)
Current breastfeeding or breastfed until 6 months of age					
Yes	592 (85.6)	536 (77.6)	544 (80.7)	586 (82.7)	319 (89.1)
No	100 (14.5)	155 (22.4)	130 (19.3)	123 (17.4)	39 (10.9)
Current breastfeeding					
Yes	404 (58.4)	345 (49.9)	345 (51.2)	375 (52.9)	215 (60.1)
No	288 (41.6)	346 (50.1)	329 (48.8)	334 (47.1)	143 (39.9)
**Medical problems**					
Congenital disorder(s)					
Yes	7 (1.0)	9 (1.3)	2 (0.3)	8 (1.1)	4 (1.1)
No	685 (99.0)	682 (98.7)	672 (99.7)	701 (98.9)	354 (98.9)
Underlying illness(es)					
Yes	8 (1.2)	12 (1.7)	10 (1.5)	21 (3.0)	7 (2.0)
No	684 (98.8)	679 (98.3)	664 (98.5)	688 (97.0)	351 (98.0)
Ever hospitalized					
Yes	125 (18.1)	159 (23.0)	152 (22.6)	182 (25.7)	80 (22.4)
No	567 (81.9)	532 (77.0)	522 (77.5)	527 (74.3)	278 (77.7)
Cough in the preceeding two weeks					
Yes	311 (44.9)	300 (43.4)	328 (48.7)	345 (48.7)	155 (43.3)
No	381 (55.1)	391 (56.6)	346 (51.3)	364 (51.3)	203 (56.7)
Runny nose in the preceeding two weeks					
Yes	336 (48.6)	340 (49.2)	364 (54.0)	382 (53.9)	175 (48.9)
No	356 (51.5)	351 (50.8)	310 (46.0)	327 (46.1)	183 (51.1)
Difficult breathing in the preceeding two weeks					
Yes	16 (2.3)	18 (2.6)	29 (4.3)	23 (3.2)	20 (5.6)
No	676 (97.7)	673 (97.4)	645 (95.7)	686 (96.8)	338 (94.4)
Have taken antibiotics in the preceeding two weeks					
Yes	132 (19.1)	172 (24.9)	148 (22.0)	182 (25.7)	112 (31.3)
No	560 (80.9)	519 (75.1)	526 (78.0)	527 (74.3)	246 (68.7)

**Vaccination history**					
BCG					
Yes	690 (99.7)	691 (100.0)	669 (99.3)	708 (99.9)	355 (99.2)
No	2 (0.3)	0 (0.0)	5 (0.7)	1 (0.1)	3 (0.8)
DPT					
4 doses	65 (9.4)	112 (16.2)	105 (15.6)	111 (15.7)	43 (12)
3 doses	568 (82.1)	519 (75.1)	516 (76.6)	542 (76.5)	292 (81.6)
2 doses	45 (6.5)	48 (7.0)	42 (6.2)	42 (5.9)	19 (5.3)
1 dose	12 (1.7)	10 (1.5)	11 (1.6)	10 (1.4)	2 (0.6)
No	2 (0.3)	2 (0.3)	0 (0)	4 (0.6)	2 (0.6)
Measles					
2 doses	75 (10.8)	125 (18.1)	131 (19.4)	124 (17.5)	58 (16.2)
1 dose	347 (50.1)	310 (44.9)	315 (46.7)	319 (45)	161 (45)
No	270 (39)	256 (37.1)	228 (33.8)	266 (37.5)	139 (38.8)
PCV					
4 doses	1 (0.1)	0 (0.0)	0 (0.0)	3 (0.4)	0 (0.0)
3 doses	0 (0.0)	2 (0.3)	0 (0.0)	2 (0.3)	0 (0.0)
2 doses	3 (0.4)	6 (0.9)	4 (0.6)	3 (0.4)	2 (0.6)
1 dose	3 (0.4)	2 (0.3)	1 (0.2)	6 (0.9)	2 (0.6)
No	685 (99.0)	681 (98.6)	669 (99.3)	695 (98.0)	354 (98.9)
**Socio-economic status**					
Number of household members					
Median (interquartile range)	5 (4-6)	5 (4-7)	5 (4-7)	5 (4-6)	5 (4-6)
People density in household (people/100m^2)					
Median (interquartile range)	7.1 (5.0-10.0)	6.7 (4.7-10.0)	7.1 (5.0-10.0)	7.0 (4.7-10.0)	5.8 (4.7-10.0)
Usually in company with child(ren) <5 years old					
Yes	493 (71.2)	522 (75.5)	509 (75.5)	590 (83.2)	280 (78.2)
No	199 (28.8)	167 (24.2)	165 (24.5)	119 (16.8)	78 (21.8)
Unknown					
Ever attended day-care/kindergarten					
Yes	166 (24.0)	188 (27.2)	200 (29.7)	194 (27.4)	100 (27.9)
No	526 (76.0)	503 (72.8)	474 (70.3)	515 (72.6)	258 (72.1)
Currently attend day-care/kindergarden					
Yes	158 (22.8)	180 (26.1)	194 (28.8)	187 (26.4)	98 (27.4)
No	534 (77.2)	511 (74.0)	480 (71.2)	522 (73.6)	260 (72.6)
Smoker(s) in household					
Indoor smoker(s)	129 (18.6)	147 (21.3)	151 (22.4)	106 (15)	104 (29.1)
Outdoor only smoker(s)	278 (40.2)	297 (43.0)	285 (42.3)	273 (38.5)	111 (31.0)
No smoker	285 (41.2)	247 (35.8)	238 (35.3)	330 (46.5)	143 (39.9)
Have farm animal(s)					
Yes	91 (13.2)	55 (8.0)	94 (14.0)	77 (10.9)	74 (20.7)
No	601 (86.9)	636 (92)	580 (86.1)	632 (89.1)	284 (79.3)
Household income last month (one million Vietnamese dong)					
Median (interquartile range)	10 (7-15)	10 (8-15)	10 (8-15)	10 (8-20)	10 (7-15)
Highest education level in household					
No school/primary	178 (25.7)	180 (26.1)	167 (24.8)	95 (13.4)	115 (32.1)
Secondary	162 (23.4)	148 (21.4)	182 (27.0)	168 (23.7)	76 (21.2)
High school	185 (26.7)	202 (29.2)	194 (28.8)	203 (28.6)	102 (28.5)
College/university	167 (24.1)	161 (23.3)	131 (19.4)	243 (34.3)	65 (18.2)
Mother's education level in household					
No school/primary	70 (10.1)	34 (4.9)	78 (11.6)	34 (4.8)	38 (10.6)
Secondary	157 (22.7)	162 (23.4)	155 (23.0)	144 (20.3)	68 (19.0)
High school	208 (30.1)	224 (32.4)	208 (30.9)	215 (30.3)	131 (36.6)
College/university	257 (37.1)	271 (39.2)	233 (34.6)	316 (44.6)	121 (33.8)
**Pneumococcal carriage** (n=3123)					
Pneumococcal carriage					
Yes	211 (30.5)	187 (27.1)	218 (32.3)	209 (29.5)	128 (35.8)
No	481 (69.5)	503 (72.9)	456 (67.7)	500 (70.5)	230 (64.3)
PCV10-type pneumococcal carriage					
Yes	95 (13.7)	92 (13.3)	111 (16.5)	108 (15.2)	55 (15.4)
No	597 (86.3)	598 (86.7)	563 (83.5)	601 (84.8)	303 (84.6)

Table S2: Proportion of children completing the specified schedule for age group and trial arm in the final cross-sectional survey (October 2020). For infants, this represents finishing the specified primary series and for toddlers, this represents finishing both the specified primary series + booster.

Age group	Schedule	Specified number of doses for age group and schedule	Received under the specified number of doses of PCV10 (%, x/N)	Received the specified number of doses of PCV10 (%, x/N)	Received over the specified number of doses of PCV10 (%, x/N)	Received at least the specified number of doses of PCV10 (%, x/N)
Infants	Unvaccinated	0	0%* (0/178)	82.6%* (147/178)	17.4%* (31/178)	100.% (178/178)
	0p+1	0	0%* (0/317)	76.7%* (243/317)	23.3%* (74/317)	100.% (317/317)
	1p+1	1	5.09% (17/334)	85.9% (287/334)	8.98% (30/334)	94.9% (317/334)
	2p+1	2	17.9% (61/341)	77.1% (263/341)	4.99% (17/341)	82.1% (280/341)
	3p+0	3	22.3% (70/314)	77.1% (242/314)	0.637% (2/314)	77.7% (244/314)
Toddlers	Unvaccinated	0	0%* (0/183)	83.1%* (152/183)	16.9%* (31/183)	100.% (183/183)
	0p+1	1	10.2% (37/361)	65.9% (238/361)	23.8% (86/361)	89.8% (324/361)
	1p+1	2	13.4% (47/351)	79.2% (278/351)	7.41% (26/351)	86.6% (304/351)
	2p+1	3	20.0% (70/350)	76.0% (266/350)	4.00% (14/350)	80.0% (280/350)
	3p+0	3	29.3% (100/341)	66.6% (227/341)	4.11% (14/341)	70.7% (241/341)

*specified to receive 0 doses

Table S3: Non-inferiority of reduced dose schedules (A: 1p+1, B: 0p+1) versus standard dose schedules (2p+1 and 3p+0) by age group (infants: 4-11 months, toddlers: 14-24 months) by mean and 95% confidence intervals (CI) of the difference in absolute prevalence in the penultimate carriage survey in 2019 for PCV-10 and PCV-10 + serotype 6A.

Age group	Vaccine types	Standard dose schedule	Reduced dose schedule	Standard dose VT prevalence (%, n/N)	Reduced dose VT prevalence (%, n/N)	Absolute difference in VT carriage prevalence (percentage points, 95% CI)
Infants	PCV10	2p+1	0p+1	1.5 (5/340)	3.8 (12/316)	2.3 (-0.1, 4.8)
		3p+0		1.3 (4/313)	3.8 (12/316)	2.5 (0.1, 5)
		2p+1	1p+1	1.5 (5/340)	1.8 (6/333)	0.3 (-1.6, 2.2)
		3p+0		1.3 (4/313)	1.8 (6/333)	0.5 (-1.4, 2.4)
	PCV10 + 6A	2p+1	0p+1	4.4 (15/340)	8.5 (27/316)	4.1 (0.4, 7.9)*
		3p+0		2.2 (7/313)	8.5 (27/316)	6.3 (2.8, 9.8)*
		2p+1	1p+1	4.4 (15/340)	5.4 (18/333)	1 (-2.3, 4.3)
		3p+0		2.2 (7/313)	5.4 (18/333)	3.2 (0.2, 6.1)*
Toddlers	PCV10	2p+1	0p+1	3.8 (13/343)	2.5 (9/360)	-1.3 (-3.9, 1.3)
		3p+0		1.8 (6/340)	2.5 (9/360)	0.7 (-1.4, 2.9)
		2p+1	1p+1	3.8 (13/343)	3.7 (13/347)	0 (-2.9, 2.8)
		3p+0		1.8 (6/340)	3.7 (13/347)	2 (-0.5, 4.4)
	PCV10 + 6A	2p+1	0p+1	11.7 (40/343)	10.8 (39/360)	-0.8 (-5.5, 3.8)
		3p+0		7.1 (24/340)	10.8 (39/360)	3.8 (-0.4, 8)*
		2p+1	1p+1	11.7 (40/343)	8.4 (29/347)	-3.3 (-7.8, 1.2)
		3p+0		7.1 (24/340)	8.4 (29/347)	1.3 (-2.7, 5.3)*

The * refers to comparisons which cross the non-inferiority margin of >5% difference in the upper bound of the 96% confidence interval.

Supplementary Figures

Figure S1: Non-inferiority results using 2019 vaccine type pneumococcal carriages.

Figure S2: Proportion of pneumococcal serotype carriage at baseline 2016 and 2020.

Table S4: PCV Reduced Dosing Schedules study subjects Severe Adverse Events list

No.	Sex	Age(mo)	Vaccination Date	PCV dose	SAE	Date of onset	Hospitalization date	Final diagnosis	Outcome	Notes
1	M	17	2017/2/15	1st	Hospitalization (vomitting, cough)	2017/2/15	2017/2/16	Bronchitis and gastroenteritis	Recovered without sequelae	
2	F	26	2017/2/17	1st	Hospitalization (lower limb weakness)	2017/2/25	2017/2/25	Reactive arthritis	Recovered without sequelae	
3	M	6	2017/2/17	1st	Hospitalization (fever)	2017/2/17	2017/2/17	Fever after vaccination	Recovered without sequelae	
4	M	14	2017/2/20	1st	Hospitalization (fever, hoarse voice)	2017/2/20	2017/2/21	Laryngitis and bronchitis	Recovered without sequelae	
5	M	8	2017/2/21	1st	Hospitalization (fever)	2017/2/21	2017/2/21	Fever after vaccination	Recovered without sequelae	
6	M	33	2017/2/23	1st	Hospitalization (fever, rash)	2017/2/24	2017/2/24	Allergy	Recovered without sequelae	
7	F	2	2017/3/6	1st	Hospitalization (fever)	2017/3/6	2017/3/6	Fever after vaccination	Recovered without sequelae	
8	F	3	2017/4/10	1st	Hospitalization (fever)	2017/4/10	2017/4/10	Fever after vaccination	Recovered without sequelae	
9	M	5	2017/4/18	2nd	Hospitalization (fever, vomitting)	2017/4/18	2017/4/18	Gastroenteritis	Recovered without sequelae	
10	M	2	2017/9/7	1st	Hospitalization (fever)	2017/9/7	2017/9/7	Fever after vaccination	Recovered without sequelae	
11	M	3	2018/1/3	2nd	Hospitalization (fever, cough, seizure)	2018/1/3	2018/1/3	Pneumonia	Recovered without sequelae	
12	M	2	2018/3/6	1st	Hospitalization (fever)	2018/3/6	2018/3/6	Fever after vaccination	Recovered without sequelae	
13	F	5	2018/6/4	2nd	Hospitalization (fever, right thigh swelling and pain)	2018/6/4	2018/6/4	Fever after vaccination	Recovered without sequelae	PCV10 given to left thigh, other EPI vaccine given to right thigh
14	F	3	2018/6/6	1st	Hospitalization (fever)	2018/6/6	2018/6/7	Fever after vaccination	Recovered without sequelae	
15	F	2	2018/7/2	1st	Hospitalization (fever, seizure, syanosis)	2018/7/2	2018/7/2	Side effects of vaccination	Recovered without sequelae	
16	M	3	2018/12/4	1st	Hospitalization (fever)	2018/12/4	2018/12/4	Fever after vaccination	Recovered without sequelae	
17	F	4	2019/1/2	3rd	Hospitalization (fever)	2019/1/2	2019/1/2	Fever after vaccination	Recovered without sequelae	
18	F	12	2019/1/2	1st	Hospitalization (fever, rash, itchiness)	2019/1/2	2019/1/2	Vaccine allergy	Recovered without sequelae	
19	F	2	2019/1/7	1st	Hospitalization (fever, rash)	2019/1/7	2019/1/7	Fever after vaccination	Recovered without sequelae	
20	M	2	2019/2/13	1st	Hospitalization (fever)	2019/2/13	2019/2/13	Fever after vaccination	Recovered without sequelae	
21	F	3	2019/3/1	1st	Hospitalization (fever, vomitting, cough, stuffy nose)	2019/3/1	2019/3/1	Upper respiratory infection	Recovered without sequelae	
22	F	6	2019/3/4	2nd	Hospitalization (fever)	2019/3/4	2019/3/4	Fever after vaccination	Recovered without sequelae	
23	F	3	2019/3/5	1st	Hospitalization (fever)	2019/3/5	2019/3/5	Fever after vaccination	Recovered without sequelae	
24	M	12	2019/3/6	3rd	Hospitalization (fever, cough)	2019/3/10	2019/3/11	Upper respiratory infection	Recovered without sequelae	
25	F	6	2019/3/15	3rd	Hospitalization (fever)	2019/3/15	2019/3/15	Fever after vaccination	Recovered without sequelae	
26	M	4	2019/4/3	1st	Hospitalization (fever, inflammation of right thigh)	2019/4/3	2019/4/3	Fever after vaccination, Right thigh muscle inflammation	Recovered without sequelae	PCV10 given to left thigh, other EPI vaccine given to right thigh
27	M	2	2019/4/3	1st	Hospitalization (fever, cough, runny nose, syanosis)	2019/4/3	2019/4/3	Fever after vaccination, Acute rhinitis	Recovered without sequelae	
28	M	2	2019/4/3	1st	Hospitalization (fever, vomitting)	2019/4/3	2019/4/3	Fever after vaccination	Recovered without sequelae	
29	F	4	2019/4/3	1st	Hospitalization (fever, right thigh swelling)	2019/4/3	2019/4/3	Right thigh soft tissue inflammation after vaccination	Recovered without sequelae	PCV10 given to left thigh, other EPI vaccine given to right thigh
30	F	12	2019/4/3	2nd	Hospitalization (fever, cough, runny nose, poor appetite)	2019/4/10	2019/4/11	Tonsillitis	Recovered without sequelae	
31	F	12	2019/4/8	3rd	Hospitalization (fever, vomitting, diarrhea)	2019/4/11	2019/4/11	Acute gastroenteritis	Recovered without sequelae	
32	M	4	2019/4/15	1st	Hospitalization (fever)	2019/4/15	2019/4/15	Fever after vaccination	Recovered without sequelae	
33	M	4	2019/4/15	1st	Hospitalization (fever)	2019/4/15	2019/4/15	Fever after vaccination	Recovered without sequelae	
34	F	12	2019/4/15	2nd	Hospitalization (fever, vomitting, cough, runny nose)	2019/4/16	2019/4/16	Pneumonia	Recovered without sequelae	
35	F	5	2019/5/4	1st	Hospitalization (fever, cough)	2019/5/7	2019/5/7	Fever after vaccination	Recovered without sequelae	
36	M	14	2019/5/14	1st	Hospitalization (fever, vomitting, cough, runny nose, seizure)	2019/5/17	2019/5/17	Upper respiratory infection, Digestive	Recovered without sequelae	
37	F	4	2019/6/4	2nd	Hospitalization (fever, diarrhea)	2019/6/4	2019/6/6	Bronchitis and pharyngitis	Recovered without sequelae	
38	M	2	2019/7/3	1st	Hospitalization (fever)	2019/7/3	2019/7/3	Fever after vaccination	Recovered without sequelae	
39	F	2	2019/7/3	1st	Hospitalization (fever, shaking hands)	2019/7/3	2019/7/3	Fever after vaccination	Recovered without sequelae	
40	M	12	2019/7/3	3rd	Hospitalization (fever, seizure)	2019/7/3	2019/7/3	Pneumonia	Recovered without sequelae	
41	F	3	2019/7/5	1st	Hospitalization (fever, shaking hands)	2019/7/5	2019/7/5	Fever after vaccination, pharyngitis	Recovered without sequelae	
42	M	3	2019/8/5	1st	Hospitalization (fever, cough)	2019/8/5	2019/8/5	Fever after vaccination	Recovered without sequelae	
43	M	12	2019/9/4	2nd	Hospitalization (fever, rash)	2019/9/4	2019/9/5	Fever after vaccination, rash	Recovered without sequelae	
44	F	6	2019/9/5	1st	Hospitalization (fever, convulsion)	2019/9/5	2019/9/5	Fever after vaccination	Recovered without sequelae	Diagnosed with epilepcy before vaccination
45	M	11	2019/9/9	2nd	Hospitalization (fever, vomitting)	2019/9/10	2019/9/12	Acute pharyngitis	Recovered without sequelae	
46	M	12	2019/11/4	3rd	Hospitalization (vomitting, diarrhea)	2019/11/4	2019/11/4	Acute enteritis	Recovered without sequelae	
47	M	12	2019/11/4	3rd	Hospitalization (fever, convulsion)	2019/11/4	2019/11/4	Fever after vaccination	Recovered without sequelae	
48	M	2	2019/11/4	1st	Hospitalization (fever)	2019/11/4	2019/11/5	Fever after vaccination	Recovered without sequelae	
49	F	4	2019/11/5	1st	Hospitalization (vomitting)	2019/11/10	2019/11/10	Milk allergy	Recovered without sequelae	The child had a same symptom two months before vaccination

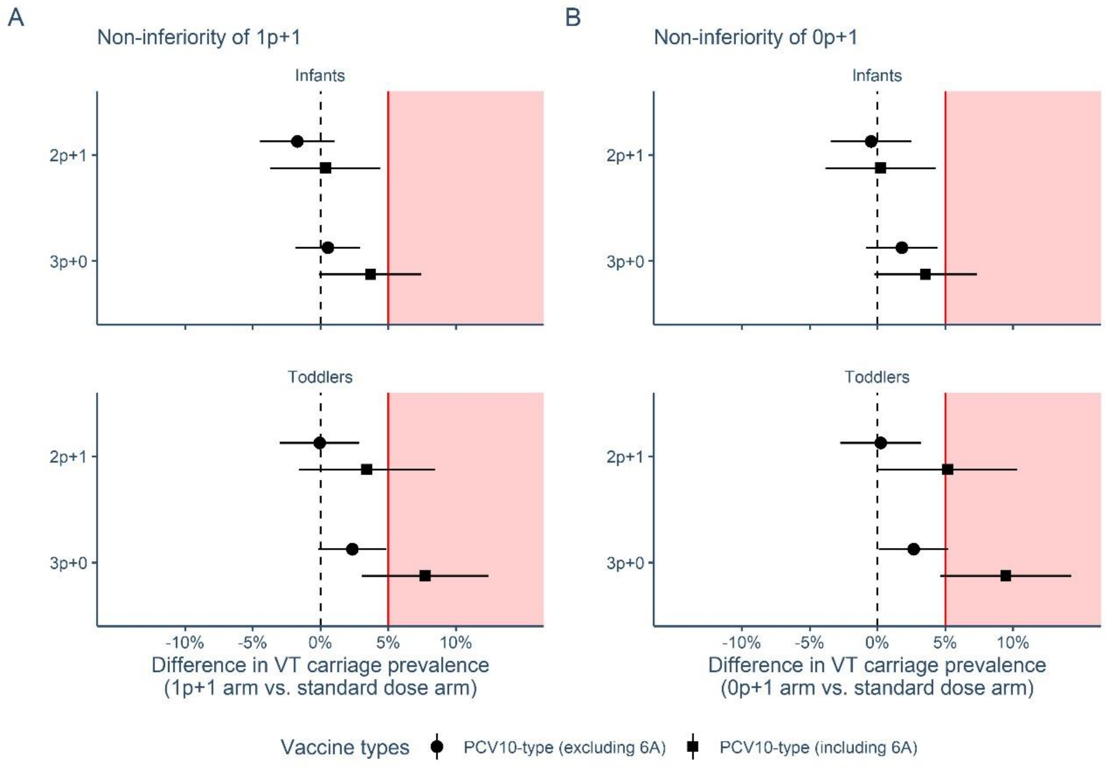

Figure S1: Non-inferiority results using 2019 vaccine type pneumococcal carriages.

Shown are the non-inferiority of reduced dose schedules (Panel A: 1p+1, and Panel B: 0p+1) versus standard dose schedules (2p+1 and 3p+0) by age group (infants: 4-11 months, toddlers: 14-24 months) by mean (point) and 95% confidence intervals (CI) (line) of the difference in absolute vaccine-type prevalence in the penultimate carriage survey in October 2019 for PCV-10 and PCV-10 + serotype 6A. Red line and shaded area indicate the 5% non-inferiority margin; estimates with 95% CIs overlapping the 5% margin indicate inferiority.

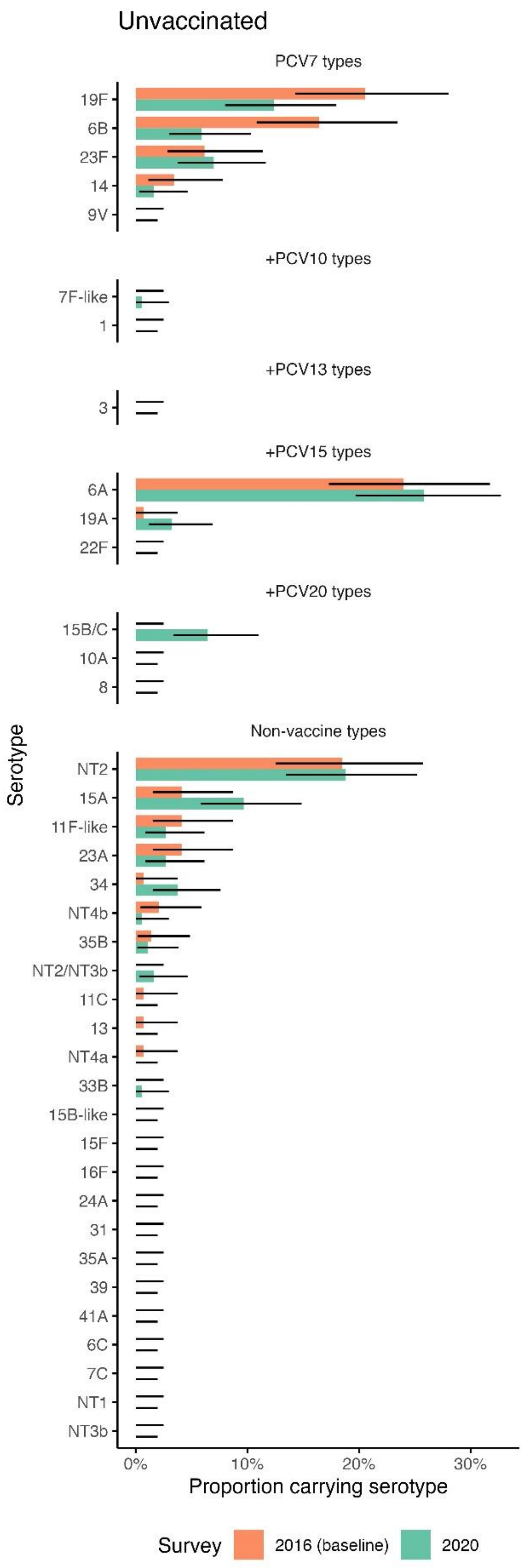

Figure S2.A

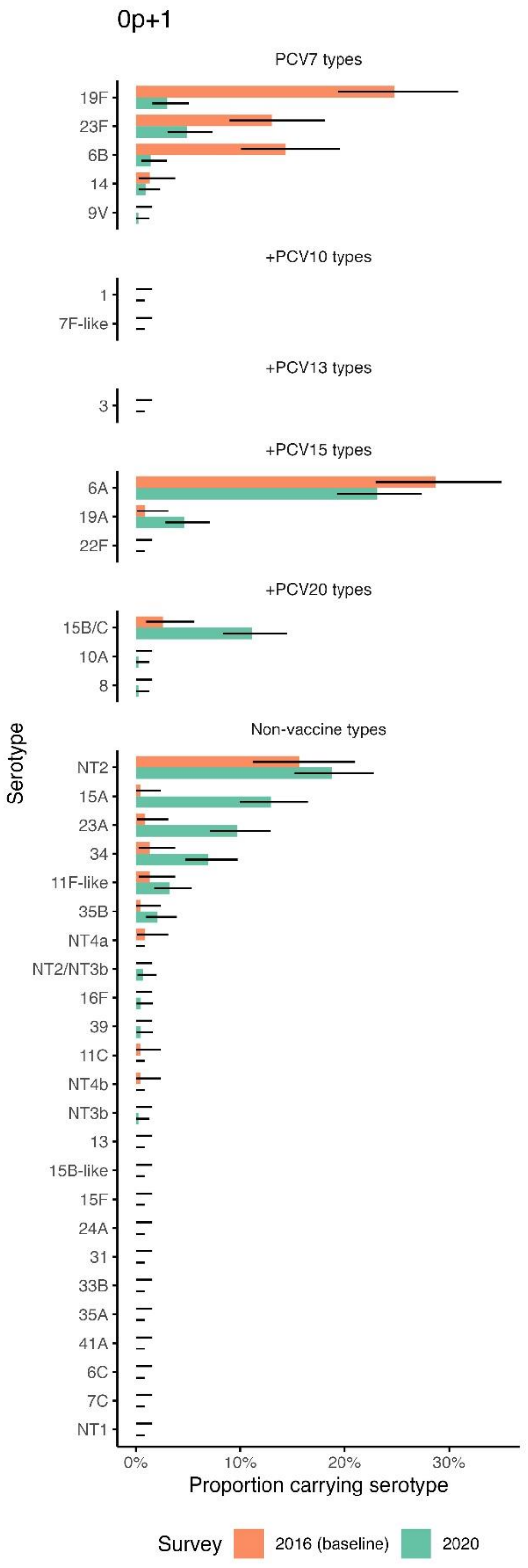

Figure S2.B

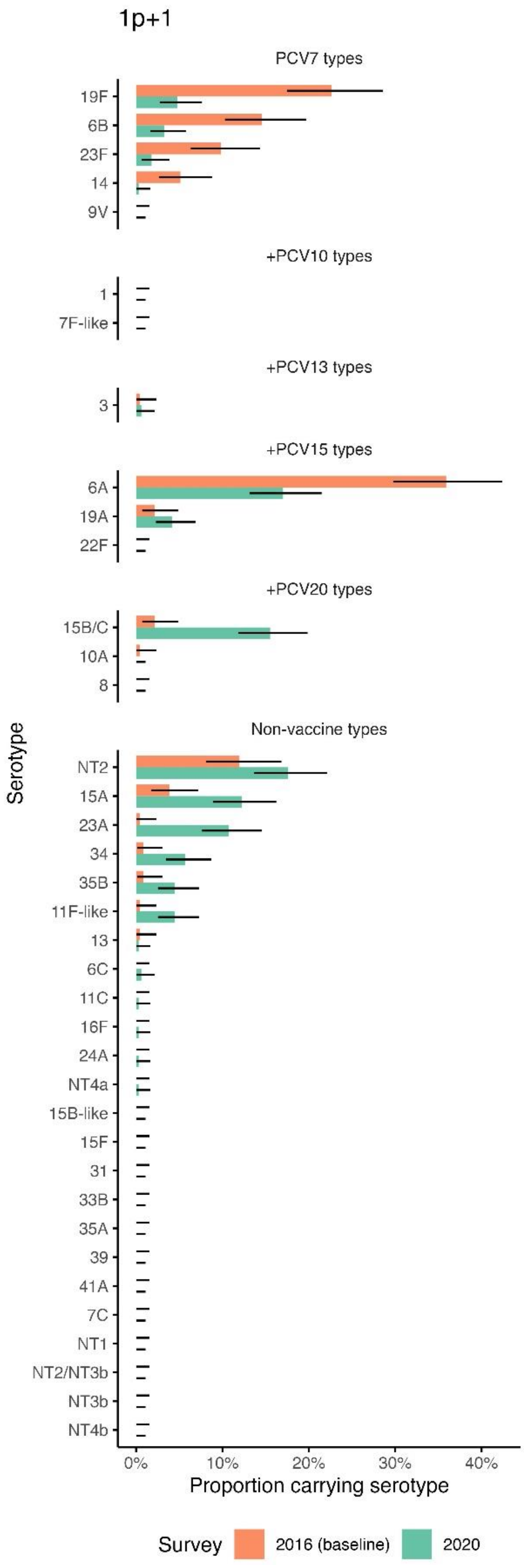

Figure S2.C

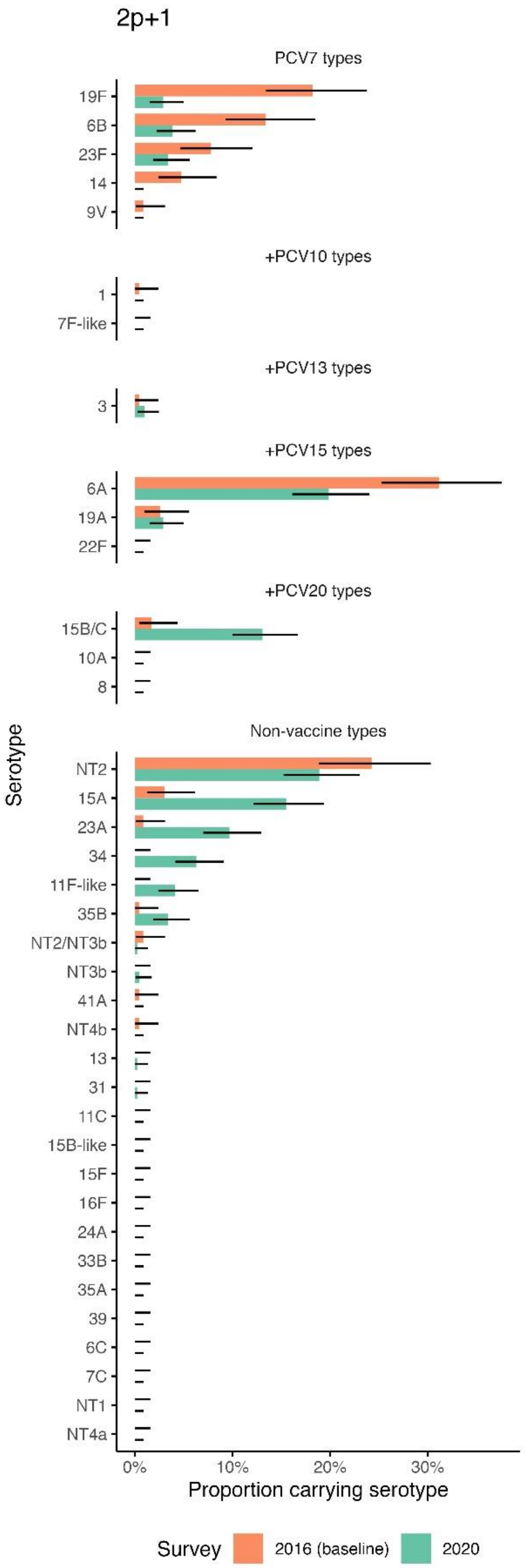

Figure S2.D

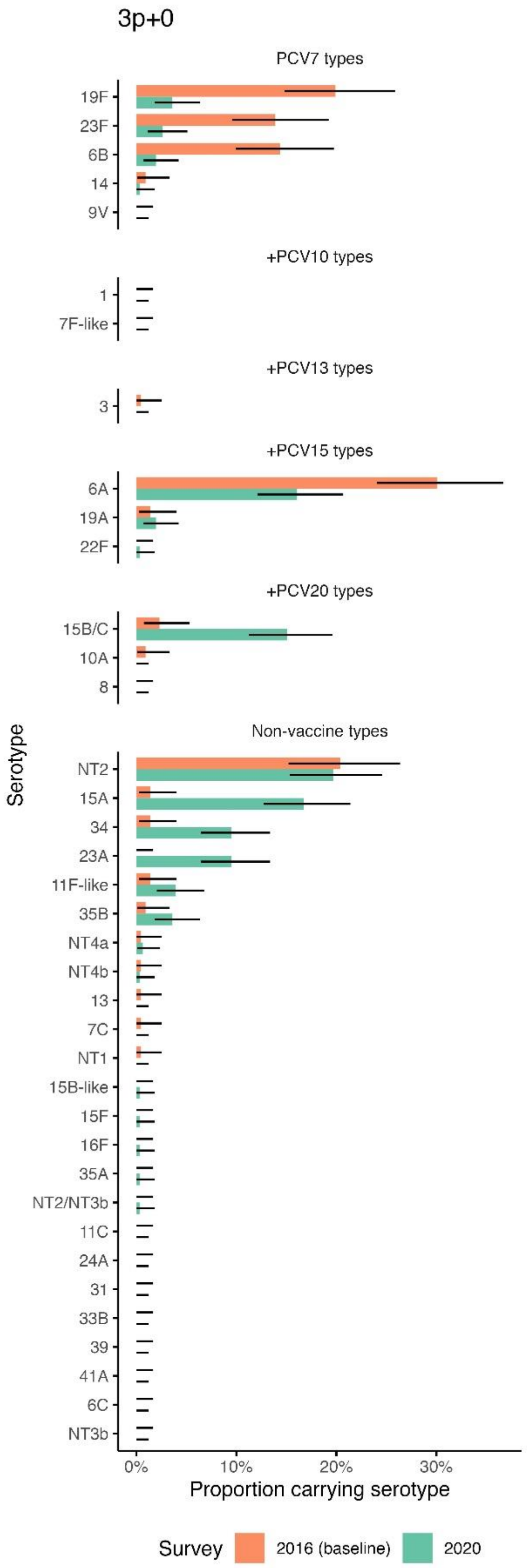
3p+0
PCV7 types
19F
23F
6B
14
9V
+PCV10 types
1
7F-like
+PCV13 types
3
+PCV15 types
6A
19A
22F
+PCV20 types
15B/C
10A
8
Non-vaccine types
NT2
15A
34
23A
11F-like
35B
NT4a
NT4b
13
7C
NT1
15B-like
15F
16F
35A
NT2/NT3b
11C
24A
31
33B
39
41A
6C
NT3b
Serotype
0%
10%
20%
30%
Proportion carrying serotype
Survey
2016 (baseline)
2020

Figure S2.E

Figure S2: Proportion of pneumococcal serotype carriage at baseline 2016 and 2020 (A. unvaccinated, B.0p+1, C. 1p+1, D. 2p+1, E. 3p+0 arms). Shown are changes in the proportion of carriers carrying a given serotype, by trial arm and inclusion in different PCV formulations, between 2016 (green bar) and 2020 (orange bar).
